# Role of Major Salivary Gland MRI in Adult Sjögren's Disease: Protocols, Findings, and Clinical Utility

**DOI:** 10.7759/cureus.110104

**Published:** 2026-06-02

**Authors:** Guilherme A Metzner, Jorge Elias

**Affiliations:** 1 Department of Medical Imaging, Hematology, and Oncology, Ribeirão Preto Medical School, University of São Paulo, Ribeirão Preto, BRA

**Keywords:** diffusion-weighted imaging (dwi), magnetic resonance imaging, major salivary gland, mr sialography, proton density fat fraction, sjögren’s disease, diagnostic imaging

## Abstract

The diagnosis of Sjögren’s disease (SD) relies on expert opinion and fulfillment of items in classification criteria sets, together with additional clinical, pathological, and laboratory evaluations. Imaging is not yet part of any criteria set, although sonography has recently demonstrated strong potential for inclusion as an additional item. Besides sonography, some evidence suggests that magnetic resonance (MR) imaging could also be a useful adjunct in diagnosing this disease. We describe and illustrate the findings of SD on conventional pulse sequences and MR sialography, as well as comment on their diagnostic performance. The role of advanced imaging techniques, including diffusion-weighted imaging (DWI) and proton-density fat fraction (PDFF) imaging, and evidence regarding how MR relates to other frequently requested complementary tests are also reviewed. We conclude by presenting several knowledge gaps that, in our opinion, need to be addressed before MR imaging can be included in the diagnostic workup of SD.

## Introduction and background

Sjögren’s disease (SD) is a systemic autoimmune disorder mainly affecting the salivary and lacrimal glands. No cure is available to this day, and long-standing disease causes a significant loss of quality of life.

Though the judgment of an experienced clinician is desirable and, to an extent, imperative in order to establish a diagnosis, classification criteria may aid in this task, the most recent of which was jointly published by the American College of Rheumatology (ACR) and the European League Against Rheumatism (EULAR) in 2016 [1]. Although non-invasive evaluation of structural damage to the salivary glands through imaging is not yet contemplated by this set of criteria, recent developments by the Outcome Measures in Rheumatology (OMERACT) group have renewed expectations that ultrasound (US) be incorporated as an additional item due to its reliability and good diagnostic performance [2]. A study by Mossel et al., for example, found a positive predictive value for the diagnosis of SD of up to 97% for the combination of abnormal US and positivity for anti-Ro antibodies [3]. Furthermore, a meta-analysis conducted by Martins et al. found a specificity of 89% for US, very close to that of minor salivary gland biopsy [4].

Nevertheless, US may have some shortcomings that merit consideration. First, most scoring systems described to this day, including the one proposed by the OMERACT group in 2019, take a semiquantitative approach, thereby rendering sonographic evaluation prone to inter-observer variability. In its original publication, the kappa coefficient of the OMERACT scoring system ranged from 0.29 to 0.85 [2]. In a later study by Schmidt et al., the same system attained a mean kappa of 0.54 [5].

Second, despite encouraging findings regarding accuracy, US evaluation may yield false-negative results in some situations. For example, Baldini et al., Cornec et al., and Lee et al. found US to have sensitivities of 66%, 65.8%, and 77.1%, respectively, in individuals with a short duration of sicca symptoms (i.e., less than five years) [6-8].

In this regard, magnetic resonance (MR) imaging could be an addition to, or even a replacement for, US in adverse situations. Among its advantages, well-adjusted MR protocols can be quick, do not require contrast medium, and allow multiparametric evaluation that can provide quantitative information through diffusion-weighted imaging (DWI) as well as proton density fat fraction (PDFF) techniques, thus potentially overcoming challenges related to inter-observer reliability and diagnosis in the early stages of disease.

**Figure 1:**
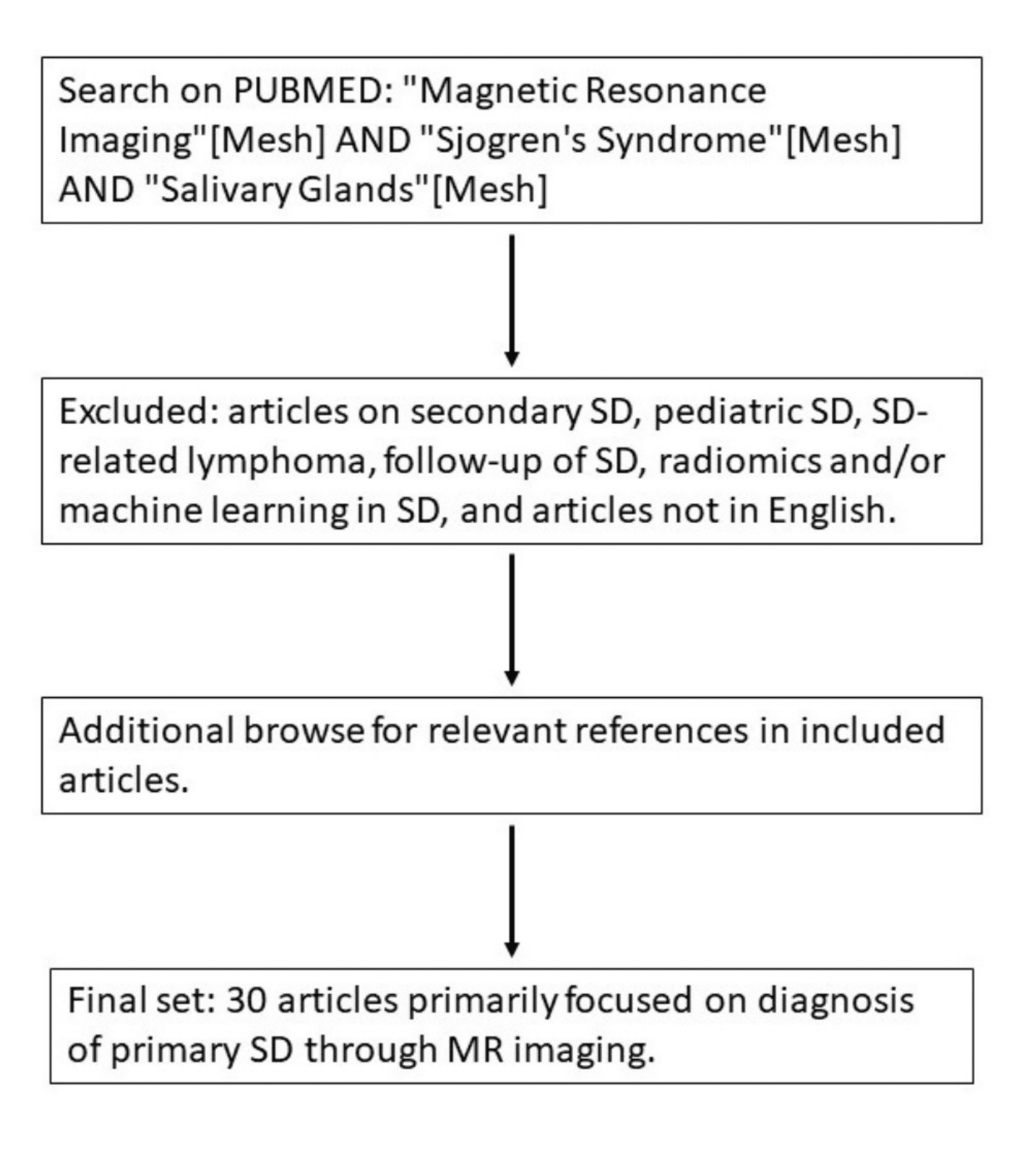
Search strategy on PUBMED. SD: Sjögren’s disease; MR: magnetic resonance

Aiming to stimulate further research, we present a concise narrative review on the role of MR in the diagnosis of SD in adults. We searched PubMed for papers on the utility of MR imaging of the major salivary glands for diagnosing primary SD, published until June 2025 and focusing on conventional pulse sequences, MR sialography, DWI, and PDFF techniques. The MeSH terms “Sjögren Syndrome,” “magnetic resonance imaging,” and “salivary glands” were added to the search code. Results were restricted to studies published in English, and we also browsed relevant references in the selected articles. Figure 1 summarizes the search strategy.

The following paper is organized as a series of questions relevant to clinicians and radiologists. We also include representative images from examinations performed at our institution. Pediatric SD, SD-related lymphoma, and the role of MR in follow-up are beyond the scope of this review. We conclude by highlighting key knowledge gaps that, in our view, demand further research to support the broader adoption of MR as a diagnostic tool for adult SD.

## Review

What are the suitable pulse sequences and the findings of SD on MR?

Conventional Pulse Sequences (T1 and T2 Weighting)

On conventional pulse sequences, normal salivary glands present with intermediate signal intensity on T1-weighted images, intermediate-to-high signal intensity on T2-weighted images, and a homogeneous texture, excluding small lymph nodes and the retromandibular vein in the parotid gland (PG) [9].

Conventional T1- and T2-weighted imaging has been explored for over 30 years in SD, in which the glands acquire an inhomogeneous texture due to T1-hyperintense, nodular-like lesions that become increasingly confluent as the disease progresses, an appearance that has been described as “salt and pepper” or “honeycomb” [9-11]. Such lesions exhibit signal loss on fat-suppressed pulse sequences, an observation that motivated Izumi et al. to propose that this textural heterogeneity occurs due to fat deposition in the salivary glands [12,13].

**Figure 2:**
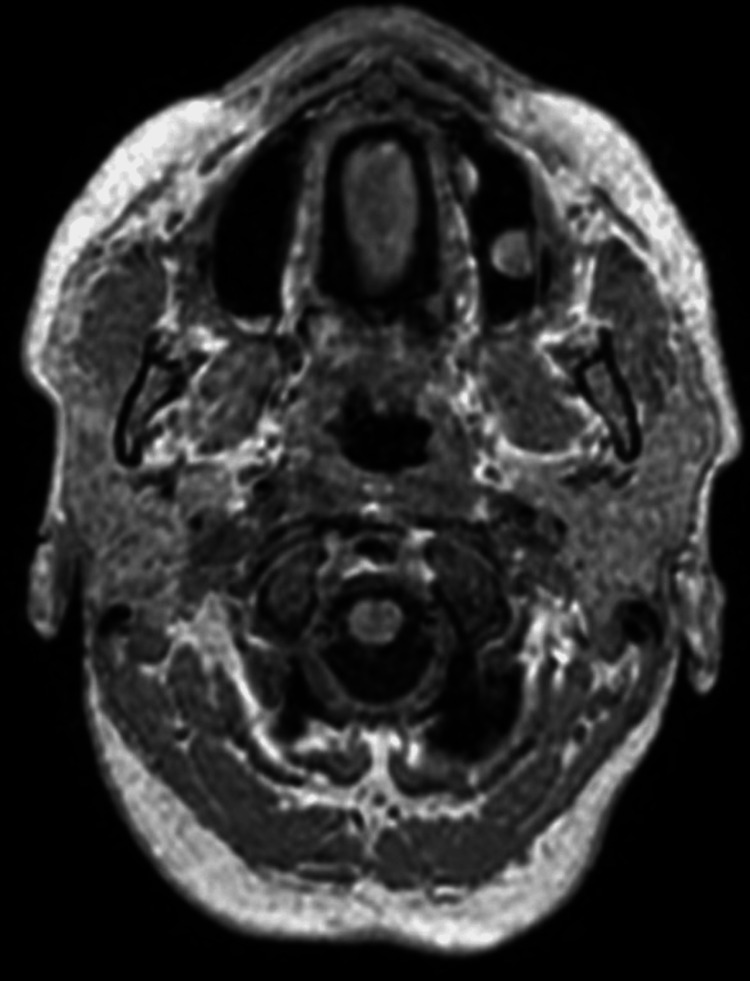
T1-weighted image depicts uniform signal, normal shape, no fat signal, and clear boundary with the surrounding tissue. Staging system for conventional imaging of the parotid glands proposed by Rao et al. [14], grade 0.

**Figure 3:**
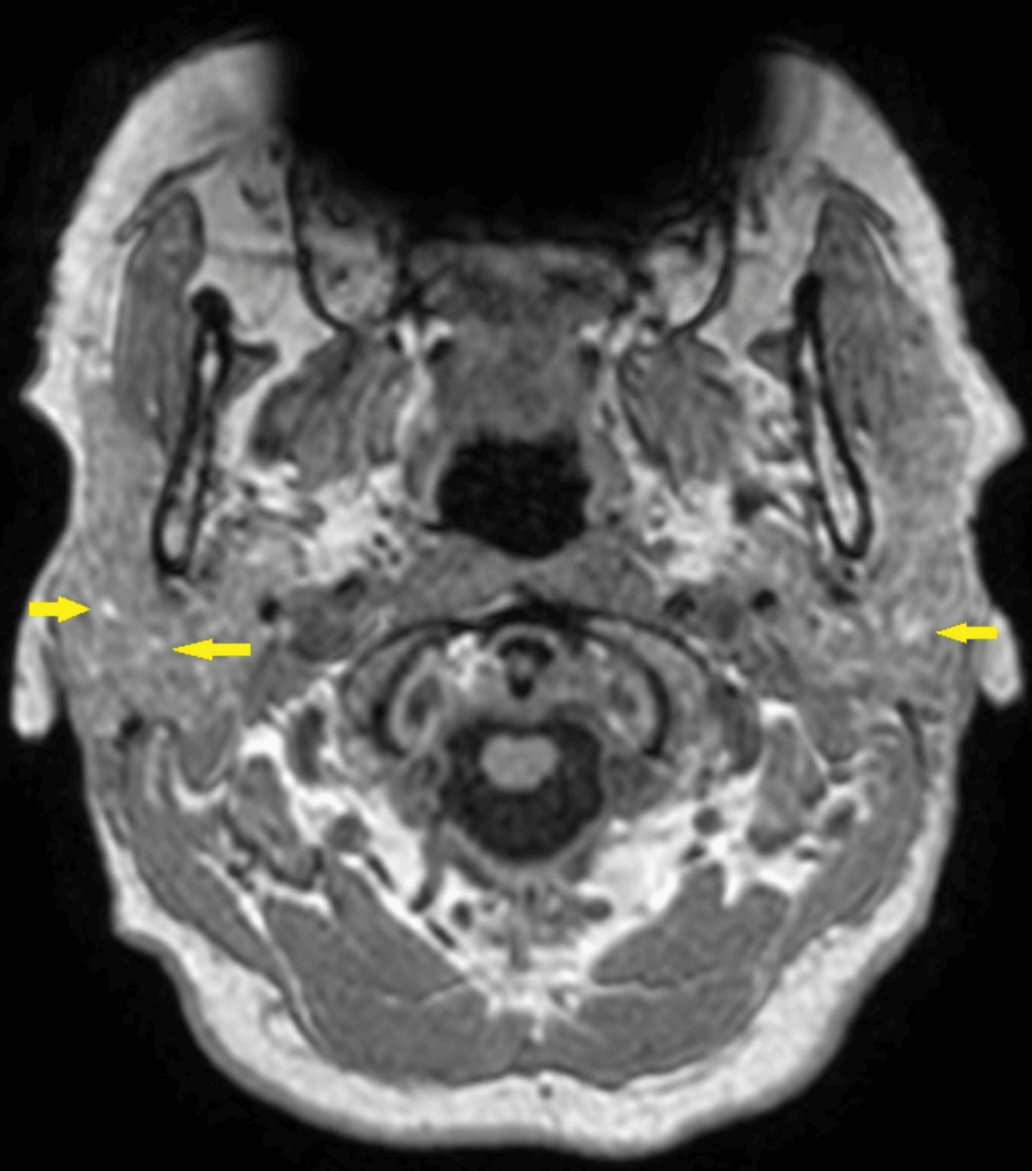
T1-weighted image depicts uneven distribution of speckled fat signal (yellow arrows) in the parotid glands. Staging system for conventional imaging of the parotid glands proposed by Rao et al. [14], grade 1.

**Figure 4:**
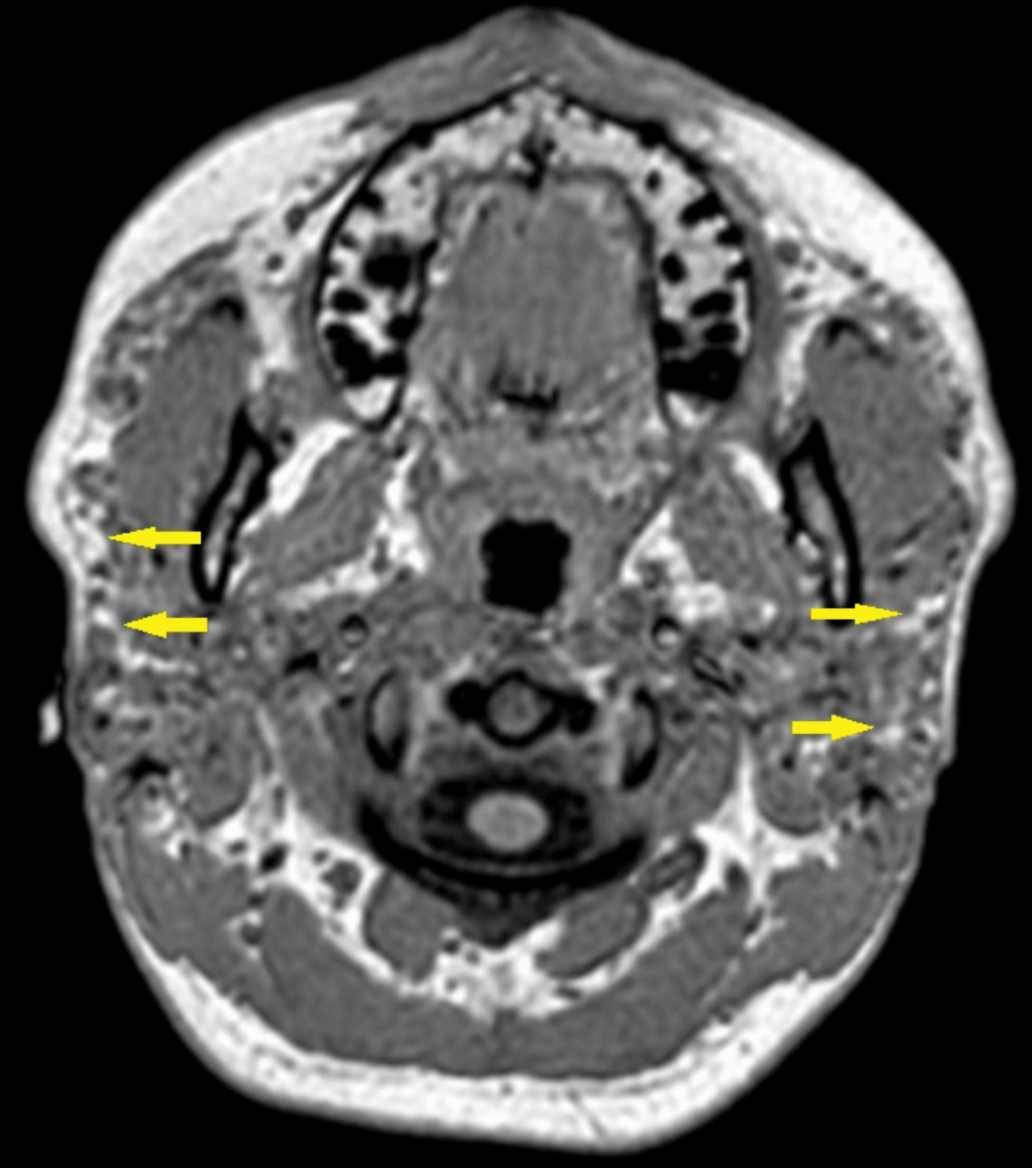
T1-weighted image depicts grid-like fat signals (yellow arrows), best seen on the right parotid gland. Staging system for conventional imaging of the parotid glands proposed by Rao et al. [14], grade 2.

**Figure 5:**
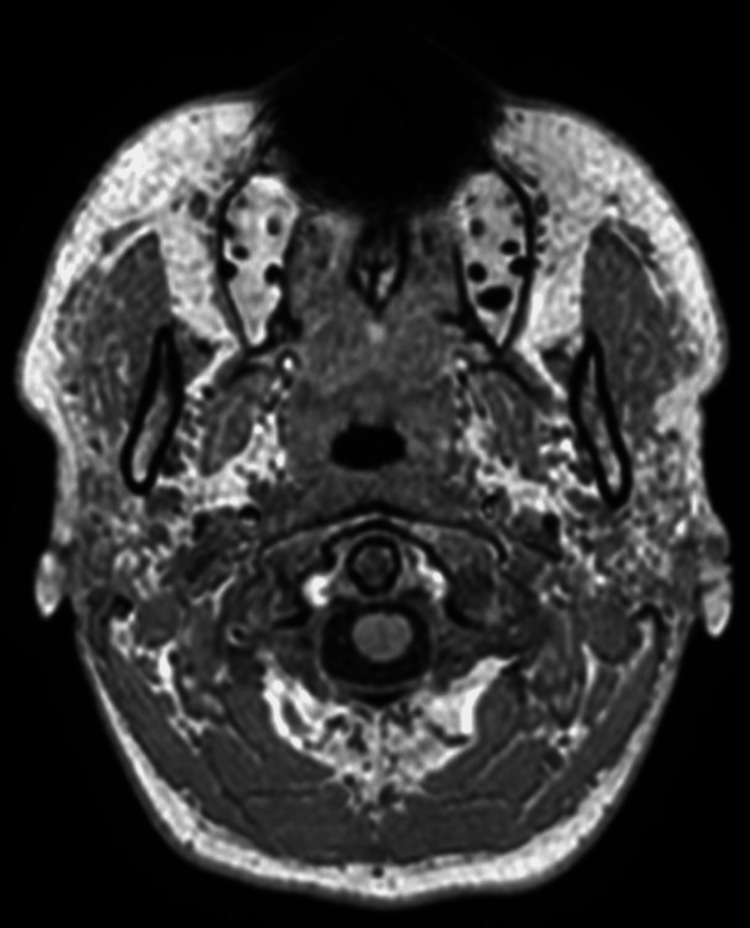
T1-weighted image depicts diffuse patch-like fat signals and residual glandular tissue with a nodule-like appearance, notably on the left parotid. Staging system for conventional imaging of the parotid glands proposed by Rao et al. [14], grade 3.

**Figure 6:**
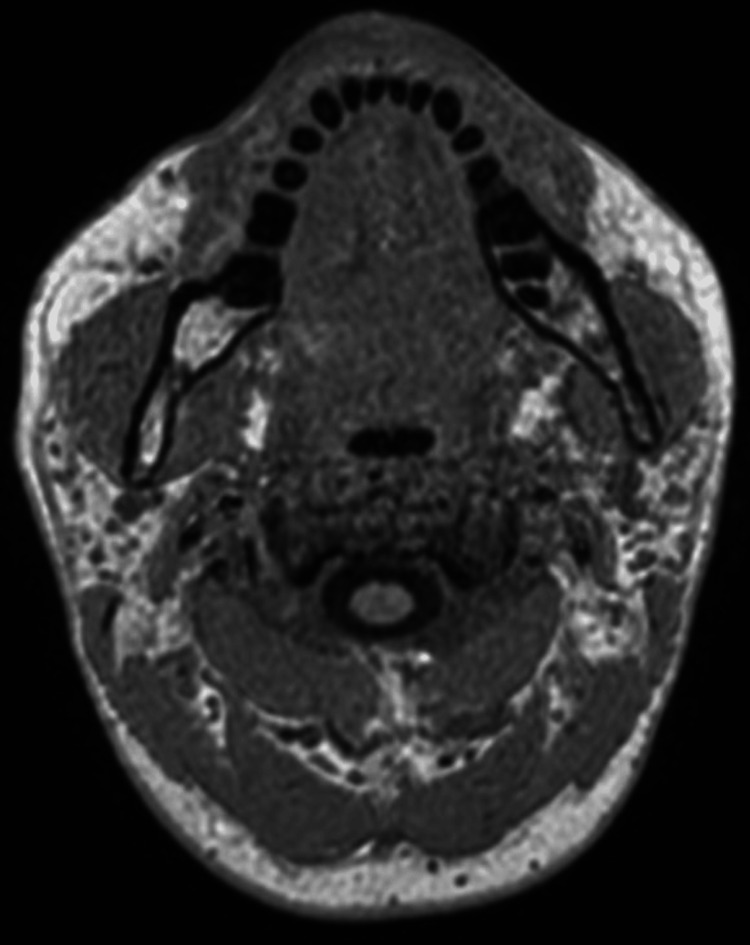
T1-weighted image depicts a large area of fat signal, with almost no normal gland. Staging system for conventional imaging of the parotid glands proposed by Rao et al. [14], grade 4.

Several staging systems for conventional pulse sequences have been proposed, as listed in Table 1 [9,11,12,14-16]. Figures 2-6 illustrate the staging system proposed by Rao et al. in 2023 [14].

**Table 1:** Proposed staging systems for conventional MR imaging of the salivary glands. MR: magnetic resonance

Author	Gland	Staging system
Izumi et al. (1996) [12]	Parotid	Grade 0: homogeneous intensity distribution on T1-weighted images and no focal high-intensity areas on T2-weighted images.
		Grade 1: sparse distribution of focal high-intensity areas on T1-weighted images and no focal high-intensity areas on T2-weighted images.
		Grade 2: sparse distribution of focal high-intensity areas on T1-weighted images and the presence of focal high-intensity areas on T2-weighted images.
		Grade 3: moderate distribution of focal high-intensity areas on T1-weighted images and the presence of focal high-intensity areas on T2-weighted images.
		Grade 4: diffuse or homogeneous distribution of high-intensity areas on T1-weighted images and no focal high-intensity areas on T2-weighted images.
Makula et al. (2000) [9]	Parotid	Grade 0: homogeneous parenchyma (normal).
		Grade 1: fine reticular or small nodular structure (nodules <2 mm).
		Grade 2: medium nodular structure (nodules 2-5 mm).
		Grade 3: coarsely nodular structure (nodules >5 mm).
Ren et al. (2015) [15]	Parotid	Grade 0: homogeneous intensity distribution without fat deposition.
		Grade 1: sparse distribution of streak-like fat signals.
		Grade 2: diffuse distribution of honeycomb-like fat signals.
		Grade 3: diffuse and patchy fat signal, occupying less than 50% of the parotid gland area.
		Grade 4: homogeneously distributed fat signal occupying more than 50% of the parotid gland area.
Kojima et al. (2019) [11]	Parotid and submandibular	Grade 0: homogeneous.
		Grade 1: almost homogeneous.
		Grade 2: slightly heterogeneous.
		Grade 3: moderately heterogeneous.
		Grade 4: severely heterogeneous or total fatty replacement.
Rao et al. (2023) [14]	Parotid	Grade 0: uniform signal, normal shape, no fat signal, and clear boundary with the surrounding tissue.
		Grade 1: uneven distribution of speckled fat signal.
		Grade 2: diffuse grid-like fat signals.
		Grade 3: diffuse patch-like fat signals, and residual glands with low-intensity nodules, with the gaps blurred.
		Grade 4: large area of fat signal, with almost no normal gland.
Cho et al. (2023) [16]	Parotid and submandibular	Grade 1: normal or sparse distribution of streak-like fat signals.
		Grade 2: diffusely distributed, honeycomb-like fat signals.
		Grade 3: less than 50% of the total area of the whole salivary glands.
		Grade 4: massively homogeneously distributed fat signal.

MR Sialography

Conventional sialography consists of the cannulation and injection of iodinated contrast media into the Stensen duct of the PGs, thereby allowing visualization of up to third-order excretory ducts due to its high spatial resolution [17]. It has historically been an item of classification criteria for SD. However, there are disadvantages to this examination, such as the requirement of manual skill for cannulation, the induction of pain upon distension of the excretory ducts, and the risk of infection [18].

Hydrographic techniques in MR imaging are most routinely applied to study the biliary tract and cerebrospinal fluid, but can also be used to investigate abnormalities in the ductal system of the salivary glands. As an advantage, MR sialography of the PGs does not require the injection of contrast media into the ductal system. Moreover, it has demonstrated 89% agreement with conventional sialography [18].

MR sialography may be implemented on a 1.5T system (e.g., Ingenia; Philips Medical Systems, Best, The Netherlands) using a heavily T2-weighted spin-echo sequence (TR 2300 ms, TE 600 ms, slice thickness 0.4 mm, matrix 376 × 242, flip angle 90°). The field of view is centered over a single PG, preferably the one with greater suspected involvement, and the source images are acquired in the sagittal plane. Acquisition can be performed both at baseline and after oral stimulation with lemon juice administered while the patient remains in the scanner; each acquisition typically requires approximately three to five minutes.

**Figure 7:**
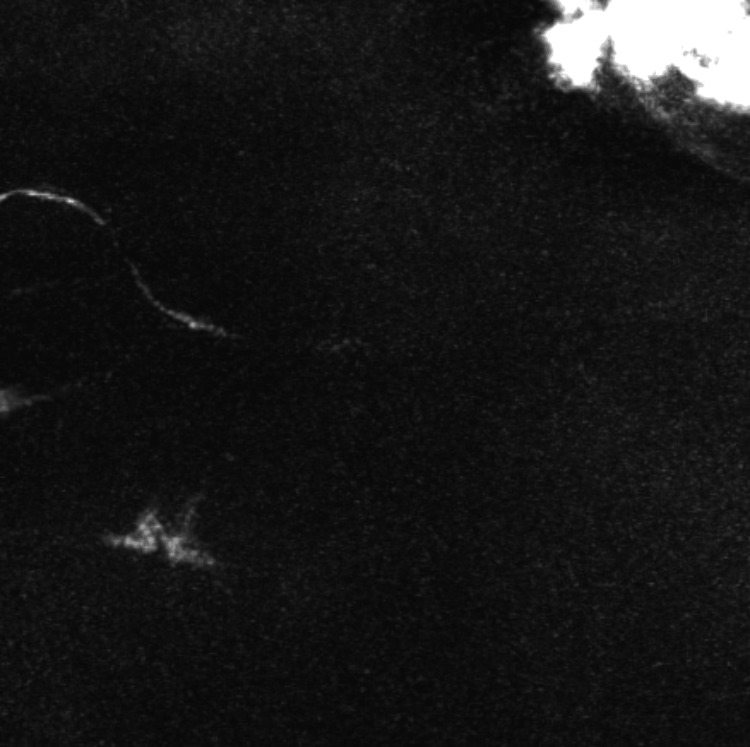
Normal main parotid duct is depicted on the left side of the image. Staging system for MR sialography of the parotid glands proposed by Tonami et al. [20], grade 0 (normal). MR: magnetic resonance

**Figure 8:**
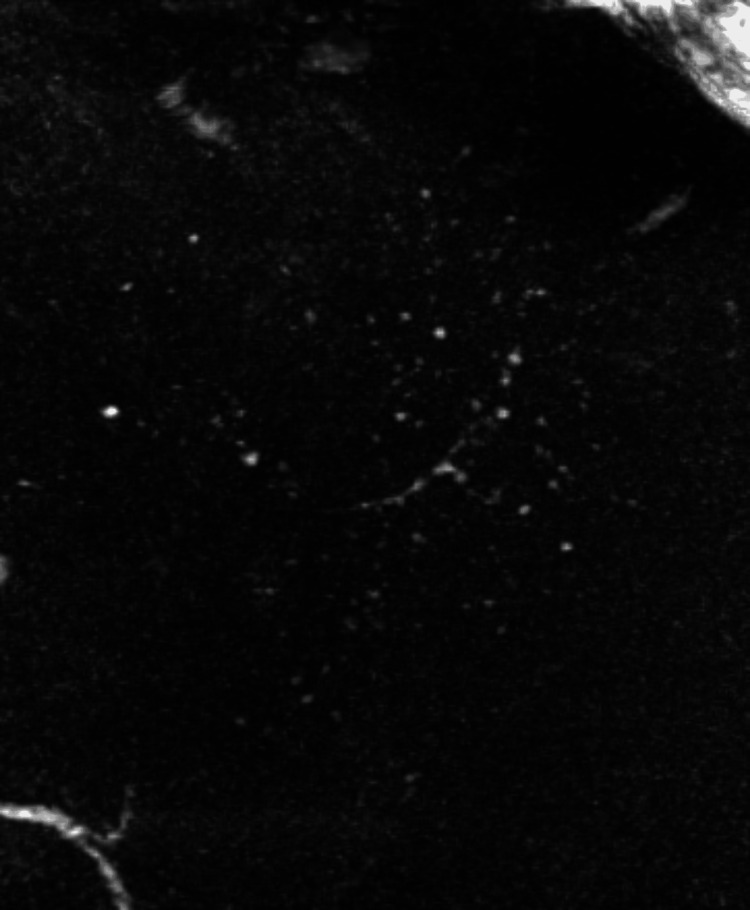
Areas of high signal intensity measuring 1 mm or less in diameter, evenly distributed throughout the gland. Staging system for MR sialography of the parotid glands proposed by Tonami et al. [20], grade 1 (punctate). MR: magnetic resonance

**Figure 9:**
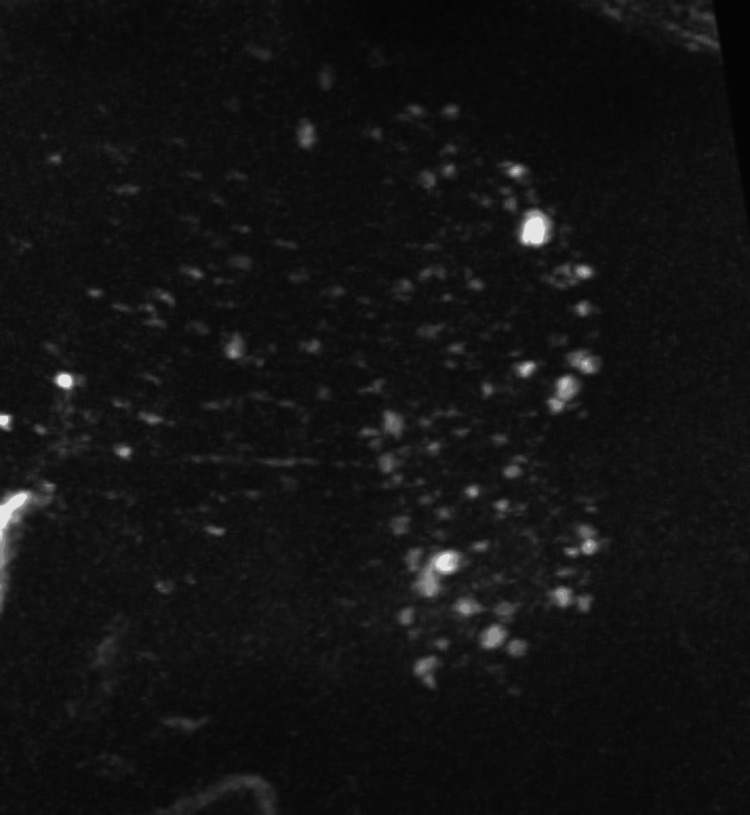
Areas of high signal intensity measuring 1 to 2 mm in diameter. Staging system for MR sialography of the parotid glands proposed by Tonami et al. [20], grade 2 (globular). MR: magnetic resonance

**Figure 10:**
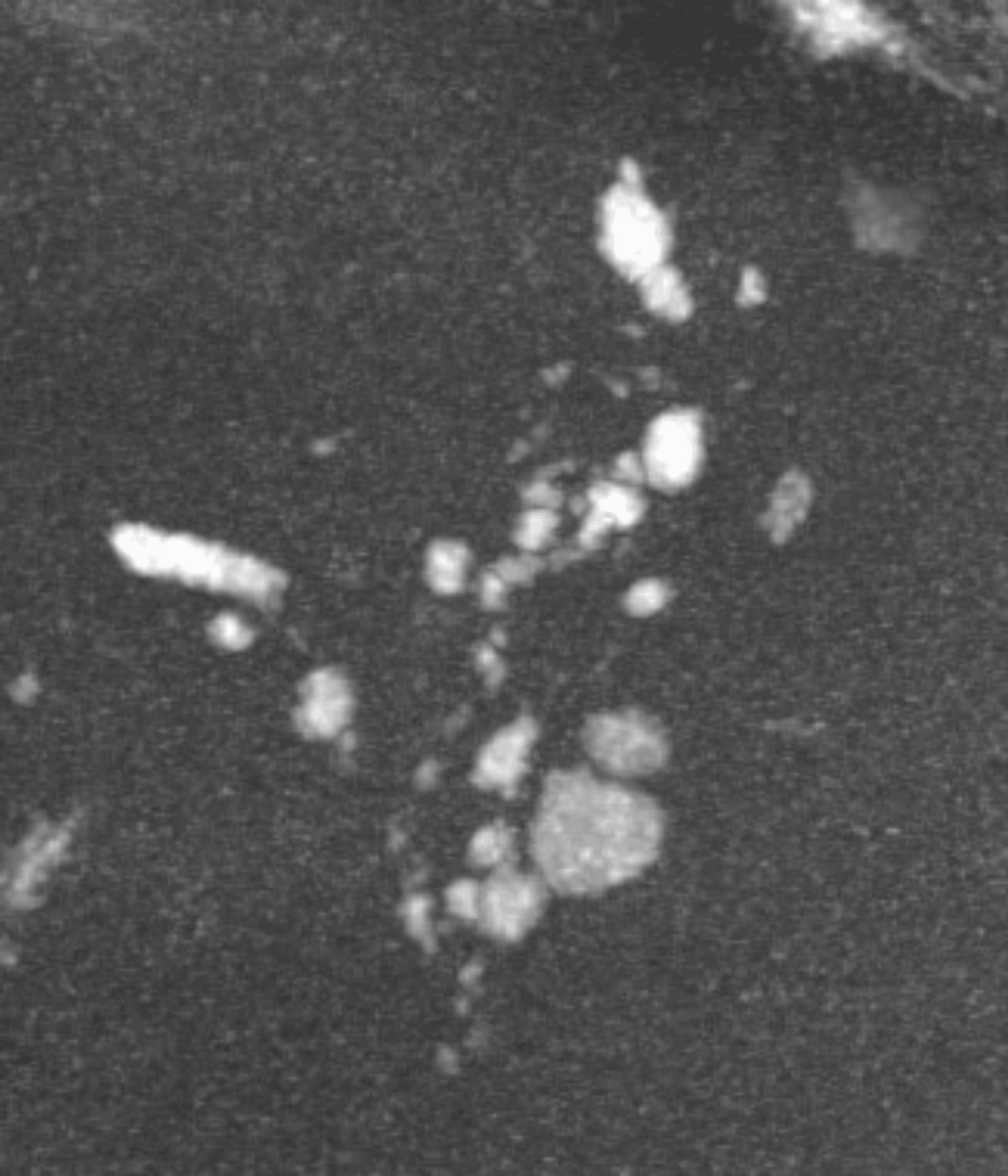
Areas of high signal intensity have coalesced and enlarged (measuring up to 1 cm of diameter), as well as become irregular in size and decreased in number. Staging system for MR sialography of the parotid glands proposed by Tonami et al. [20], grade 3 (cavitary). MR: magnetic resonance

The typical findings of MR sialography of the PGs are very similar to those of conventional sialography, namely hyperintense spots that have been referred to as “apple tree appearance” and suggested to represent cyst-like dilatations of peripheral excretory ducts (i.e., sialectasis) [19]. Tonami et al. proposed a scoring system for sialectasis in PG sialography, which is often reproduced to this day (Figures 7-10) [20]. As an additional advantage, MR sialography can also evaluate the submandibular gland (SMG) [21].

What is the diagnostic performance of MR?

The PGs have been most often studied to determine the diagnostic performance of MR. On conventional pulse sequences, their sensitivity has ranged from 67% to 81.8%, and their specificity from 75% to 100% [9,14,15,21]. We believe such wide ranges could be partly explained by the application of different scoring systems, as listed in Table 1. As for MR sialography of the PGs, its sensitivity has ranged from 68.9% to 97%, and its specificity from 83% to 100% [14,15,21-23].

Notably, Niemelä et al., Ren et al., and Rao et al. reported no association between conventional pulse sequences and MR sialography of the PGs, thus speculating that fat deposition and ductal damage do not occur simultaneously as SD progresses [14,15,22]. For that reason, an argument has been made in favor of incorporating both types of pulse sequences into SD protocols in order to increase their performance [10,22].

On an analysis emulating clinical routine, Rao et al. tested the incorporation of MR imaging of the PGs into the 2016 ACR/EULAR classification criteria [14]. When both conventional pulse sequences and MR sialography were incorporated as an additional item with a weight of 1 point, the sensitivity slightly increased (from 90.2% to 92.3%), while the specificity showed minimal change (from 89.1% to 88.9%). However, when MR imaging replaced minor salivary gland biopsy with a weight of 3 points, both sensitivity and specificity decreased (to 88.1% and 86.4%, respectively).

The SMGs have been less often studied in their ability to predict SD. Kojima et al. suggested SMG alterations to represent advanced disease due to the high specificities obtained upon their evaluation through conventional pulse sequences and MR sialography (88% and 100%, respectively), though not significantly different from the specificities obtained upon evaluation of the PGs through the same sequences (75% and 83%, respectively) [21]. In the same study, the sublingual glands were deemed inadequate for diagnosis due to low performance.

What is the place of DWI and fat fraction (FF) quantification on SD?

Diffusion-Weighted Imaging (DWI)

**Figure 11:**
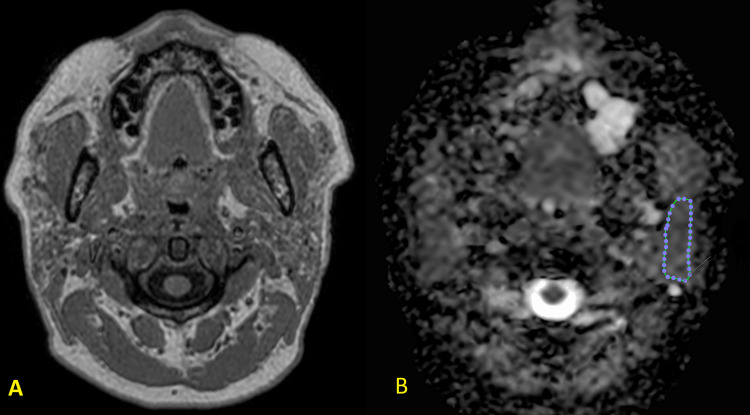
ADC measurement on the left parotid gland. (A, left) T1-weighted image depicts heterogeneity due to grid-like fat deposition in the parotid glands (Rao grade 2). (B, right) A region of interest manually drawn over the left parotid gland returned a mean ADC of 0.815 × 10⁻³ mm²/s. ADC: apparent diffusion coefficient

Important questions remain regarding the role of DWI, which explores the Brownian random movement of water molecules in the extracellular space, in the diagnosis of SD [24]. First, a consensus on the apparent diffusion coefficient (ADC) values of normal PGs has not been reached. In a 2021 review that included 43 studies [25], the reported mean ADC values for normal PGs ranged from 0.28 × 10⁻³ to 2.42 × 10⁻³ mm²/s. Second, the literature is inconsistent as to whether the ADC values in diseased glands are higher or lower than those in healthy glands. We believe such issues could be partly explained by methodological heterogeneity, for example, differences in how regions of interest (ROIs) are defined and placed within the salivary glands for ADC measurement. Below, we briefly discuss representative studies and summarize their main findings in Table 2. Figure 11 illustrates the measurement of ADC in the PG.

**Table 2:** Studies on the role of DWI in the diagnosis of SD. ADC: apparent diffusion coefficient; AUC: area under the curve; DWI: diffusion-weighted imaging; MRI: magnetic resonance imaging; PG: parotid gland; SMG: submandibular gland; ROI: region of interest; SD: Sjögren’s disease

Author	Objectives	Methods	Results
Sumi et al. (2002) [26]	Compare the ADC of the PGs and SMGs between healthy volunteers and individuals with SD.	1.5 Tesla.	The ADCs of the PGs and SMGs were lower in individuals with SD compared to normal volunteers.
		b = 500 and 1000 s/mm².	The ADC of the PGs demonstrated direct correlation with the stimulated salivary flow and inverse correlation with the Izumi scale [7] for conventional imaging.
		ROI drawn over the entire cross-sectional area of the PGs and SMGs on each slice.	-
Regier et al. (2009) [24]	Compare the ADC of the PGs between healthy volunteers and individuals with early- and advanced-stage SD.	1.5 Tesla.	Mean ADC of the PGs of healthy volunteers prior to oral stimulation = 1.14 × 10^-3 ^mm^2^/s.
	Investigate the effect of gustatory stimulus on the ADC of the PGs.	b = 0, 500, and 1000 s/mm².	Mean ADC of the PGs of early-stage SD prior to oral stimulation = 1.22 × 10^-3 ^mm^2^/s.
	-	ROI drawn over the entire cross-sectional area of the PGs on every slice.	Mean ADC of the PGs of advanced-stage SD prior to oral stimulation = 0.97 × 10^-3 ^mm^2^/s.
Ding et al. (2016) [27]	Compare methods of extracting the ADC of PGs.	1.5 Tesla.	For both methods, the ADC of individuals with SD was lower than that of healthy volunteers and individuals with non-SD xerostomia.
	Compare the ADC and DWI signal intensity of the PGs between healthy volunteers, individuals with non-SD xerostomia, and individuals with SD.	b = 0 and 1000 s/mm².	Method 1 obtained the best diagnostic performance (AUC 0.979 for DWI signal intensity and 0.790 for ADC).
	Investigate the diagnostic performance of DWI signal intensity and ADC of the PGs for diagnosing SD.	Method 1: placement of 8-10 circular ROIs over areas with high DWI signal intensity in the PGs.	-
	-	Method 2: ROI drawn over the entire cross-sectional area of the PGs on only one slice.	-
Xu et al. (2017) [28]	Compare the ADC of the PGs between healthy volunteers and individuals with SD.	3 Tesla.	For all the methods, the ADC of individuals with SD was significantly higher than that of healthy volunteers.
	Compare methods of extracting the ADC of PGs.	b = 0 and 1000 s/mm².	Method 2 obtained an intermediate AUC (0.848) for diagnosing SD compared to the other ones and was not time-consuming, thus being recommended as the ideal approach to measure ADC.
	Investigate the diagnostic performance of ADC of the PGs for diagnosing SD.	Method 1: drawing of an ROI over the entire cross-sectional area of the PG on every slice.	-
	-	Method 2: drawing of an ROI over the entire cross-sectional area of the PG on only one slice.	-
	-	Method 3: placement of three circular ROIs over the PG on only one slice.	-

Sumi et al. determined the ADCs of the PGs and SMGs to be lower in individuals with SD compared with normal volunteers [26]. Furthermore, the ADC of the PGs in their study demonstrated a direct correlation with stimulated salivary flow and an inverse correlation with the Izumi scale for conventional imaging [12].

Regier et al. measured the ADC of the entire cross-sectional area of the PGs prior to and after oral stimulation with lemon juice [24]. Prior to oral stimulation, the mean ADC of the PGs in individuals with early-stage SD was significantly higher than that of healthy volunteers, a finding hypothesized to occur due to edema and inflammation. In contrast, advanced-stage disease was characterized by significantly lower mean ADC values compared with healthy volunteers, a finding suggested to be explained by fat deposition.

Xu et al. investigated distinct ways to extract the ADC from the PGs, concluding that the placement of a single ROI comprising the entire cross-sectional area of the gland in only one slice offered the best trade-off between diagnostic performance and time needed for the task [28].

Ding et al. brought attention to the fact that most studies until then had drawn an ROI encompassing the entire cross-sectional area of the salivary glands, thereby hypothesizing that fat deposition in advanced SD could influence the ADC and affect its diagnostic performance [27]. Thus, the authors tested the exclusion of areas with fat deposition in the PGs from analysis by measuring the ADC only in areas with high signal intensity on DWI images. By using this approach, the ROC curves of the DWI signal intensity and the ADC demonstrated an area under the curve (AUC) of 0.979 and 0.790, respectively, for diagnosing SD. In comparison, when using the more straightforward method of extracting information from the entire transverse cross-sectional area of the PGs (i.e., including areas with fat deposition), the AUC of the DWI signal intensity and the ADC decreased significantly to 0.808 and 0.545, respectively. An additional finding of the same study was that both DWI signal intensity and ADC did not correlate with the scale proposed by Izumi et al. for conventional pulse sequences [12].

Intravoxel incoherent motion (IVIM) imaging is an upgrade on DWI, in which multiple b-values are applied to allow for the measurement of additional diffusion and perfusion parameters. Su et al. and Chu et al. established that this technique could be useful for diagnosing early-stage SD, namely prior to changes on conventional T1- and T2-weighted images [29,30]. Additionally, Chu et al. found both the pure diffusion coefficient (D) and perfusion fraction (f) of the PGs to inversely correlate with the Makula grading scale (r = -0.297, p = 0.019; r = -0.653, p < 0.001, respectively) [30].

Fat Fraction (FF)

Fat deposition is known to occur in the salivary glands in SD and to worsen with disease progression [14]. As previously stated, Izumi et al. proposed that the textural heterogeneity seen in conventional pulse sequences could be explained by this phenomenon [12,13]. Nowadays, a method to quantify fat in the salivary glands through MR imaging is available through PDFF techniques, which have been successfully validated against MR spectroscopy [31,32]. We believe these techniques represent an opportunity to investigate how measuring FF in the salivary glands could aid in diagnosing SD. For example, in a preliminary study with a small sample, Chikui et al. found the FF of the SMGs to differ significantly between individuals with SD and healthy volunteers (p < 0.0001) [32].

Of importance to study design, Chang et al. [33], Su et al. [34], and Lee et al. [35] demonstrated that salivary gland FF correlates significantly with body mass index (BMI) and age in healthy individuals. Therefore, such factors should be accounted for when enrolling individuals into studies on the clinical application of PDFF techniques. With this in mind, Chu et al. conducted a study on the diagnostic performance of FF in which the sample was matched for gender, age, and BMI [36]. With this careful approach, the FF of the SMGs attained an AUC of 0.927 for diagnosing SD in their study. In addition, the FF of the SMGs distinguished early-stage SD (i.e., Makula 0 on conventional weighting) from suspected SD individuals with an AUC of 0.925, thus raising questions about whether MR could attain higher sensitivity than US in this setting.

**Figure 12:**
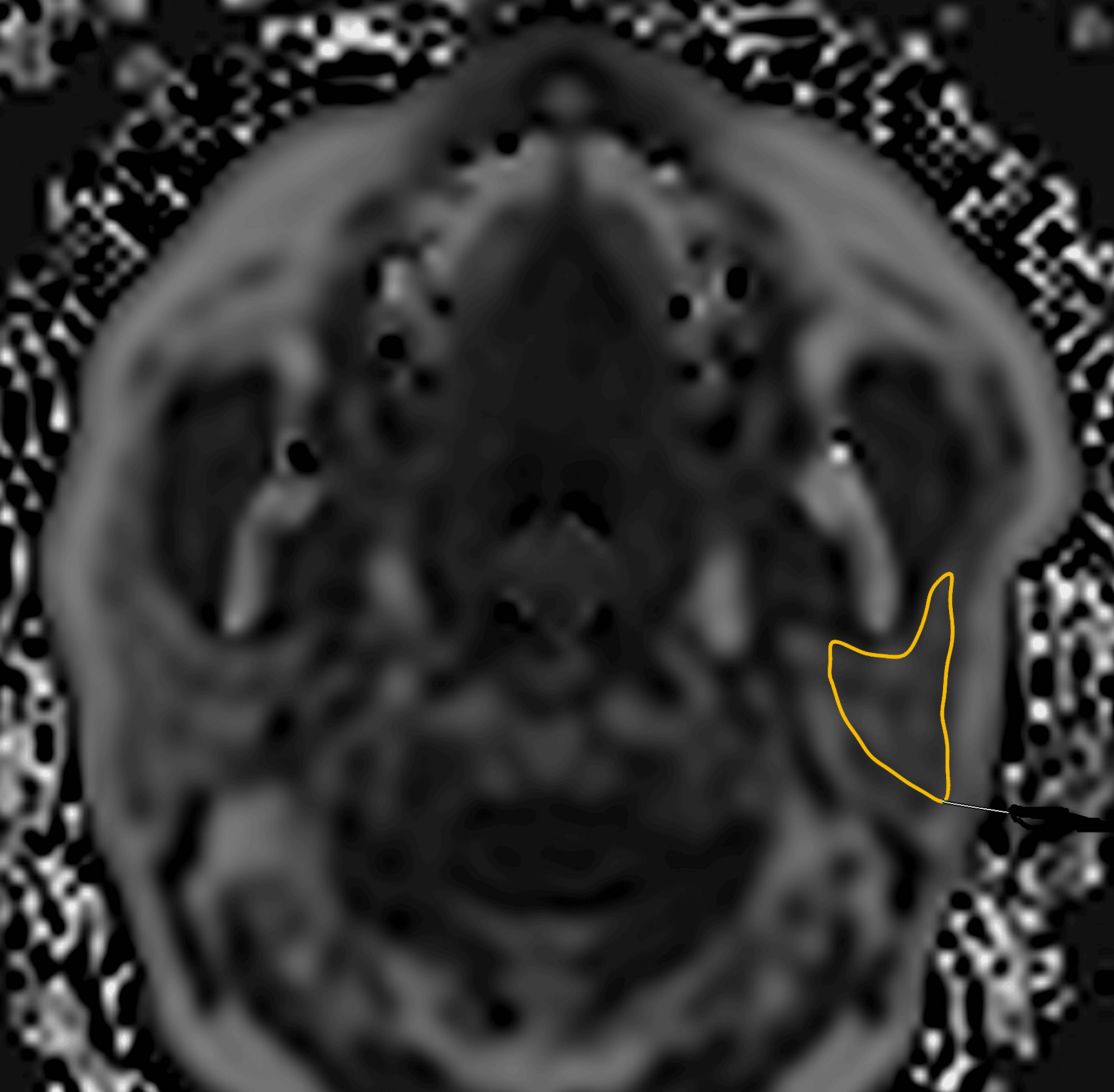
Fat fraction measurement in the left parotid gland of the same individual as in Figure 10. A manually drawn region of interest yielded a mean fat fraction of 34.8%.

Given such promising results, it is our opinion that studies on the diagnostic performance of FF should be replicated with larger samples in multiple centers. Figure 12 illustrates FF measurement in the PG.

FF quantification may be incorporated into the protocol using a multi-echo Dixon technique (e.g., mDixon Quant; Philips Medical Systems, Best, The Netherlands), acquired in the axial plane. A representative parameter set includes TR 6.59 ms, matrix 80 × 80, slice thickness 6 mm, bandwidth 2841 Hz, flip angle 5°, and echo train length 6 (TE values as per the vendor’s multi-echo acquisition scheme).

How does MR relate to US?

Few studies have directly compared MR to US. Niemelä et al. reported a correlation between US and conventional MR pulse sequences of the PGs (r = 0.76, p < 0.0001) [23]. In 44 individuals with SD, Makula et al. reported an absolute agreement of 93.2% between conventional pulse sequences and US of the PGs [9].

In 90 individuals with SD, Takagi et al. identified a correlation between the presence of hyperechogenic bands on US and fat deposition on MR in both PGs and SMGs (odds ratios 13.82 and 5.23, respectively) [19].

In 11 individuals with SD, the OMERACT scoring system for US was reported by Inanc et al. to have a moderate-to-strong agreement with a modification of the scale for conventional pulse sequences proposed by Kojima et al., which was stronger for the PGs (median 82% for the right and 91% for the left) than for the SMGs (median 55% bilaterally) [37].

How does MR relate to other tests?

Minor Salivary Gland Biopsy

Tonami et al. [20] and Rao et al. [14] reported a correlation between MR sialography of the PGs and the focus score of labial glands. On top of that, Izumi et al. [12], Niemelä et al. [23], and Rao et al. [14] reported a correlation between conventional MR imaging of the PGs and the focus score of labial glands.

In contrast, Cho et al. [16] found no correlation between MR of either PGs or SMGs (both conventional pulse sequences and sialography) and the focus score of labial glands.

Antibodies

Niemelä et al. reported an association between the findings on both MR sialography and conventional pulse sequences of the PGs and the presence of anti-Ro/SSA [22].

Cho et al. [16] found a correlation between positivity for both anti-Ro/SSA and anti-La/SSB and fat deposition in the SMGs, whereas no such correlation was identified in the PGs. Instead, the occurrence of sialectasis in the PGs correlated with positivity for anti-Ro/SSA and anti-La/SSB.

Salivary Flow

Significant associations between salivary flow rate and fat deposition in the salivary glands (evaluated through conventional MR pulse sequences) have been reported [8,11,14,19,37]. On the other hand, conflicting results regarding the ability of MR sialography to predict hyposalivation have been reported [11,14,16,22,23].

Kojima et al. identified an association between salivary flow rate and conventional imaging of both PGs and SMGs, a finding in agreement with the hypothesis that textural heterogeneity on MR imaging of the salivary glands represents fat deposition and destruction of the acini responsible for saliva production [11,13]. In contrast, no association between salivary flow rate and MR sialography of either gland was found. As for the performance of conventional imaging in predicting hyposalivation, the SMGs demonstrated a significantly higher AUC than the PGs, a finding suggested to be related to the observation that the SMGs contribute to the majority (over 65%) of resting salivary flow.

In a cohort of 11 patients with SD, Inanc et al. reported moderate-to-strong agreement between abnormalities on conventional MR and hyposalivation [37]. Agreement was higher for the PGs (median 91% bilaterally) than for the SMGs (median 73% on the right and 82% on the left).

Several other studies have also examined the relationship between MR findings and salivary output. Izumi et al. found that conventional parotid MR abnormalities were associated with stimulated salivary flow rate [13]. Takagi et al. reported correlations between conventional MRI findings and stimulated salivary flow rate for both the PG and SMG (odds ratios 36.4 and 14.73, respectively) [19]. Rao et al. observed an association between unstimulated salivary flow rate and both conventional MR and MR sialography of the PGs [14]. In contrast, Cho et al. reported that stimulated salivary flow rate correlated with fat deposition in the SMGs but not in the parotids and that parotid MR sialography did not correlate with either stimulated or unstimulated flow rates [16]. Similarly, Niemelä et al. found no correlation between parotid MR (conventional sequences and MR sialography) and unstimulated salivary flow rate in two separate reports [22,23]. Finally, Sumi et al. described an inverse relationship between parotid ADC values and stimulated salivary flow rate [26].

On a final note, Table 3 summarizes the findings of SD so far reported for each pulse sequence.

**Table 3:** Summary of imaging findings on MR imaging of salivary glands in SD. MR: magnetic resonance; SD: Sjögren’s disease; ADC: apparent diffusion coefficient

Pulse sequence	Findings reported
Conventional imaging	Textural heterogeneity due to progressive appearance and confluence of T1-hyperintense areas ("honeycomb" appearance), with signal loss on fat-suppression.
MR sialography	Progressively enlarging hyperintense spots ("apple tree" appearance).
Diffusion-weighted imaging	Conflicting findings; ADC either decreased or increased depending on disease stage.
Proton-density fat fraction imaging	Progressive fat deposition, more striking on the submandibular glands.

## Conclusions

MR can reliably depict salivary gland abnormalities associated with SD; however, we believe several areas warrant further investigation to support the incorporation of MR into the routine clinical assessment of SD. First, the SMGs should be more thoroughly investigated since they account for most of the resting salivary flow. Also, consensus scoring systems for abnormalities on conventional pulse sequences are needed to improve reproducibility. Likewise, reference values and standardized measurement approaches for quantitative parameters such as ADC and FF need to be established. Moreover, the 2016 ACR/EULAR classification criteria could be further investigated to assess their performance when MR is added as an adjunct or used to replace specific items. Finally, larger cohorts and prospective studies are necessary to understand how MR relates not only to histopathological, sonographic, and clinical features of SD but also to complementary tests (e.g., antibody titers and salivary flow rate).
